# Differential Serum Cytokine Profiles in Patients with Chronic Hepatitis B, C, and Hepatocellular Carcinoma

**DOI:** 10.1038/s41598-017-11975-7

**Published:** 2017-09-19

**Authors:** Jacqueline Estevez, Vincent L. Chen, Ondrej Podlaha, Biao Li, An Le, Philip Vutien, Ellen T. Chang, Yael Rosenberg-Hasson, Zhaoshi Jiang, Stefan Pflanz, Dongliang Ge, Anuj Gaggar, Mindie H. Nguyen

**Affiliations:** 1 Stanford University Medical Center, Division of Gastroenterology and Hepatology, Stanford, CA 94305 USA; 2 Boston University School of Medicine, Boston, MA 02118 USA; 3 Stanford University Medical Center, Department of Medicine, Stanford, CA 94305 USA; 4 University of Michigan, Division of Gastroenterology and Hepatology, Ann Arbor, MI USA; 5 Gilead Sciences, Foster City, CA 94404 USA; 6 Rush University Medical Center, Chicago, IL 60612 USA; 7 Stanford University School of Medicine, Department of Health Research and Policy (Epidemiology), Stanford, CA 94305 USA; 8 Stanford University Medical Center, The Human Immune Monitoring Center, Stanford, CA 94305 USA

## Abstract

Cytokines play an important role in the pathogenesis of cirrhosis and hepatocellular carcinoma (HCC), most cases of which are related to either hepatitis B virus (HBV) or hepatitis C virus (HCV). Prior studies have examined differences in individual cytokine levels in patients with chronic liver disease, but comprehensive cytokine profiling data across different clinical characteristics are lacking. We examined serum cytokine profiles of 411 patients with HCC (n = 102: 32% HBV, 54% HCV, 14% non-viral) and without HCC (n = 309: 39% HBV, 39% HCV, 22% non-viral). Multiplex analysis (Luminex 200 IS) was used to measure serum levels of 51 common cytokines. Random forest machine learning was used to obtain receiver operator characteristic curves and to determine individual cytokine importance using Z scores of mean fluorescence intensity for individual cytokines. Among HCC and non-HCC patients, cytokine profiles differed between HBV and HCV patients (area under curve (AUC) 0.82 for HCC, 0.90 for non-HCC). Cytokine profiles did not distinguish cirrhotic HBV patients with and without HCC (AUC 0.503) or HCV patients with and without HCC (AUC 0.63). In conclusion, patients with HBV or HCV infection, with or without HCC, have distinctly different cytokine profiles, suggesting potential differences in disease pathogenesis and/or disease characteristics.

## Introduction

Hepatocellular carcinoma (HCC) is the third most common cause of cancer-related deaths worldwide^1^, with half a million cases diagnosed annually^2^. Most cases of HCC are due to chronic infection with either hepatitis B virus (HBV) or hepatitis C virus (HCV). Globally, chronic hepatitis B is the most common cause of HCC, with highest prevalence in East Asia and sub-Saharan Africa^2^. There are approximately 248 million people with chronic hepatitis B^3^ and about 80 million people with chronic hepatitis C worldwide^4^. In the United States, one of the leading causes of cancer-related deaths is HCC^5^, and its incidence is continuing to increase^1^. Within the United States, more than 3 million people have chronic hepatitis C^1,4^, and approximately 2 million people have chronic hepatitis B^6^.

Although chronic infection with HBV (a DNA virus) or HCV (an RNA virus) leads to HCC, these two viruses differ in the pathogenic mechanism by which they cause HCC, and they result in different clinical presentations of HCC^7,8^. Given these differences, we expect the immune interaction to differ between HBV and HCV infection, and this difference should be reflected in serum cytokine and chemokine levels. The pathogenesis of HCC due to chronic liver disease involves stimulation of the immune system causing persistent inflammation and fibrosis^9^. It is hypothesized that cirrhosis and HCC occur due to the accumulation of mutations from the continuous cycle of inflammation, necrosis, regeneration, and proliferation of hepatocytes. Most cases of HCV-related HCC and non-viral HCC present with cirrhosis^10^. However, approximately 20% of HBV-related HCC cases do not present with cirrhosis, which may be due to a direct carcinogenic effect by HBV^7^. Liver samples from HCC tumor tissue and non-tumorous tissue have shown integration of HBV DNA into the host cellular DNA^8^. More importantly, this integration commonly disrupts expression of cellular genes involved in regulation of cell proliferation and viability leading to genetic alterations that can promote carcinogenesis^11^. On the other hand, HCV is an RNA virus that does not integrate into the host cellular genome. HCV likely causes HCC through the indirect pathway of chronic inflammation, similar to non-viral etiologies of HCC, and through a more direct pathway involving the interaction of HCV core protein with dendritic cells (DC). This interaction results in reduced interferon (IFN)-α and interleukin (IL)-12 levels, which interrupts the DCs’ function of priming T-cell surveillance potentially leading to HCC carcinogenesis^12,13^. Nevertheless, it is still unclear how the host immune system interacts with the virus to enhance its carcinogenic activity and how these interactions differ depending on the type of virus.

Cytokines, signaling molecules in the immune response against pathogens, likely play a key role in the pathogenesis of hepatitis, cirrhosis, and HCC. Falasca *et al*. found that HBV-infected patients had higher levels of plasma IFN-γ, tumor necrosis factor (TNF)-α, and IL-2 compared to HCV and healthy control groups. They also found that IL-6 and IL-18 were higher in both HBV and HCV groups compared to controls^14^. Another study found that liver-infiltrating T cells from chronic hepatitis C patients produced IFN-γ but not IL-4 or IL-5, while T cells from chronic hepatitis B patients were able to produce IFN-γ, IL-4, and IL-5^15^. In patients with cirrhosis and HCC, elevated expression levels of IL-6 and TNF-α were found^16,17^. Other studies done on mice and cell lines have linked IL-6 and TNF-α to HCC via interactions with signal transducer and activator of transcription 3 (STAT3) and nuclear factor kappa B (NF-κB)^18,19^. Coulouarn *et al*. found that human HCC cells expressed similar genes as mice hepatocytes treated with Transforming growth factor (TGF)-β for a short or long period of time. In this study, shorter exposure time to TGF-β activated genes associated with cell cycle arrest and apoptosis, while longer exposure time activated genes associated with invasion and metastasis^20^. Another example of a cytokine involved in HCC carcinogenesis is IL-1α. Apoptotic hepatocytes release IL-1α, which can trigger Kupffer cell-dependent compensatory proliferation of hepatocytes^21^. It is hypothesized that HCC development can occur through conversion of premalignant, oncogene-induced senescent cells. These senescent cells are known to secrete IL-6, IL-8, cutaneous T-cell attracting chemokine (CTACK), IL-1α, leptin/leptin receptor, monocyte chemoattractant protein 1 (MCP1), and regulated on activation normal T cell expressed and secreted (RANTES)^22^. Liu *et al*. previously found increased levels of IP-10 in serum and tumor tissue and reduced IP-10 receptors in peripheral blood lymphocytes in patients with HCC, which suggests that lymphocytes became desensitized to the high levels of IP-10^23^. This could be one strategy for HCC to evade the immune system.

To date, many studies of HCC have examined only a handful of cytokines or chemokines, and few have studied immune and inflammatory “profiles” based on a more comprehensive panel of immune biomarkers measured simultaneously using uniform and sensitive techniques. Our aim is to simultaneously study serum levels of 51 immune biomarkers of HCC patients with different viral etiologies and clinical characteristics using multiplex analysis of patient’s serum samples. Characterization of these immune profiles provides novel information that may help us understand the mechanistic roles cytokines and chemokines play in HCC pathogenesis in the context of different underlying liver diseases.

## Methods

### Patient Population

This is a prospective study of 411 patients with chronic liver disease who were enrolled at the liver clinics at Stanford University Medical Center between 2001 and 2010. Detailed questionnaires, administered by a study coordinator, were used to obtain risk factor information. After patients were consented, blood was drawn, and serum and plasma were processed according to standard protocols.

### Clinical Data

Patient case report forms were used to abstract medical records for demographic and clinical information. Information on diagnosis of HCC, viral hepatitis, and non-viral hepatitis, and their treatments was obtained by review of laboratory, pathological, radiological, and clinical records. Non-viral diseases included alcoholic liver disease and non-alcoholic fatty liver disease. Each liver disease was determined via serum markers for viral hepatitis, diagnostic imaging, pathology, and serum markers for HCC and cirrhosis. A risk factor questionnaire was also administered, which included 35 questions about patient demographic, past medical and surgical history, viral hepatitis risk factors, and other pertinent clinical factors relating to viral hepatitis, cirrhosis, and HCC.

### Multiplex Cytokine Assay

The FDA-approved Luminex 200 IS system was used to measure 51 serum cytokines simultaneously and work was performed by the Stanford Human Immune Monitoring Core. This multiplex system is based on flow cytometry and allows detection of up to 100 cytokines in a single 96-well plate with a sensitivity of 4 pg/ml per analyte. The cytokines compared were: cluster of differentiation 40 (CD40) ligand, Epithelial Cell-derived Neutrophil-activating Peptide-78 (ENA-78), eotaxin, fibroblast growth factor (FGF)-basic, granulocyte-colony stimulating factor (G-CSF), granulocyte macrophage CSF (GM-CSF), GRO-α, hepatocyte growth factor (HGF), intracellular adhesion molecule-1 (ICAM-1), IFN-α, IFN-β, IFN-γ, IL-10, IL-12 p40, IL-12 p70, IL-13, IL-15, IL-17A, IL-17F, IL-1α, IL-1β, IL-1RA, IL-2, IL-4, IL-5, IL-6, IL-7, IL-8, interferon gamma-induced protein-10 (IP-10), leptin, leukemia inhibitory factor (LIF), macrophage CSF (M-CSF), MCP-1, MCP-3, monocyte induced by gamma interferon (MIG), macrophage inflammatory protein (MIP)-1α, MIP-1β, nerve growth factor (NGF), plasminogen activator inhibitor-1 (PAI-1), platelet derived growth factor-BB (PDGF-BB), RANTES, resistin, stem cell factor (SCF), sFas ligand, TGF-α, TGF-β, TNF-α, TNF-β, TNF-related apoptosis-inducing ligand (TRAIL), vascular cell adhesion molecule-1 (VCAM-1), and vascular endothelial growth factor (VEGF). The assay was performed in the Human Immune Monitoring Center at Stanford University. Human 51-plex kits were purchased from Affymetrix/eBiosciences and used according to the manufacturer’s recommendations with modifications as described below. Serum from the 411 subjects were divided into two 25-μl aliquots, which were then distributed across ten 96-well plates, with both aliquots of serum from each subject placed in adjacent wells in the same plate. Each plate contained sera from some subjects with HCC and some subjects without. Each plate also contained two replicates of a “control” serum (taken from a middle-aged Caucasian male), calibration samples to aid in converting mean fluorescent intensity values to units of concentration, and custom assay control beads (CHEX1-CHEX4 by Radix Biosolutions) designed to assist in detecting experimental failures. Briefly, samples were mixed with anti-cytokine antibody-linked polystyrene beads on 96-well filter-bottom plates and incubated at room temperature for 2 h followed by overnight incubation at 4 °C. Room temperature incubation steps were performed on an orbital shaker at 500–600 rpm. Plates were vacuum-filtered and washed twice with wash buffer, and then incubated with biotinylated detection antibody for 2 h at room temperature. Samples were then filtered and washed twice as above and re-suspended in streptavidin-PE. After incubation for 40 minutes at room temperature, two additional vacuum washes were performed, and the samples re-suspended in Reading Buffer. Each sample was measured in duplicate. Plates were read using a Luminex 200 instrument with a lower bound of 100 beads per sample per cytokine. The Luminex reader identified and classified individual analytes by their bead color using the red laser, and quantifies analyte levels using the excitation of the green laser. Data are imported into BeadView software and analyzed to obtain 51 standard curves for each analyte. Data were converted to pg/ml and presented graphically or clustered into heat maps using Significance Analysis of Microarrays software (http://www-stat.stanford.edu/~tibs/SAM/). All laboratory work was performed in blinded fashion in regards to liver disease etiology and HCC status of study subjects.

This study was approved by the Institutional Review Board (IRB) at Stanford University Medical Center, and all methods were carried out in accordance with the IRB’s guidelines and regulations. All study participants gave written, informed consent.

### Analysis of Clinical Data

Stata/SE 11.1 (College Station, Texas) was used to perform all statistical analysis. A two-sided *p* value of less than 0.05 was considered statistically significant. The Student’s *t*-test was used to analyze continuous variables. These variables were reported as a mean ± standard deviation if normally distributed or as a median and range if skewed. The chi-squared test was used to analyze categorical variables. These variables were reported as a proportion (%) of the overall cohort.

### Analysis of Immune Profiles

The raw fluorescence intensity of individual cytokine measurements was Z-score transformed and used in subsequent analyses. The classification of different patient groups was performed by the randomForest package in R (version 3.2.3; www.r-project.org), where the number of trees (parameter ntree) was specified as 2000 to obtain stable results. The importance of individual cytokines was measured by Gini index during the classification; cytokines with higher mean decreased Gini index were considered to be more important than cytokines with lower numbers. All classification performance measurements, including area under the curve (AUC), accuracy, sensitivity, specificity, and precision, were calculated using the ROCR package in R. Multidimensional scaling (MDS) and Principal Component Analysis (PCA) plots were used for visual comparison of cytokine levels.

## Results

### Study Design and Patient Population

**Figure 1:**
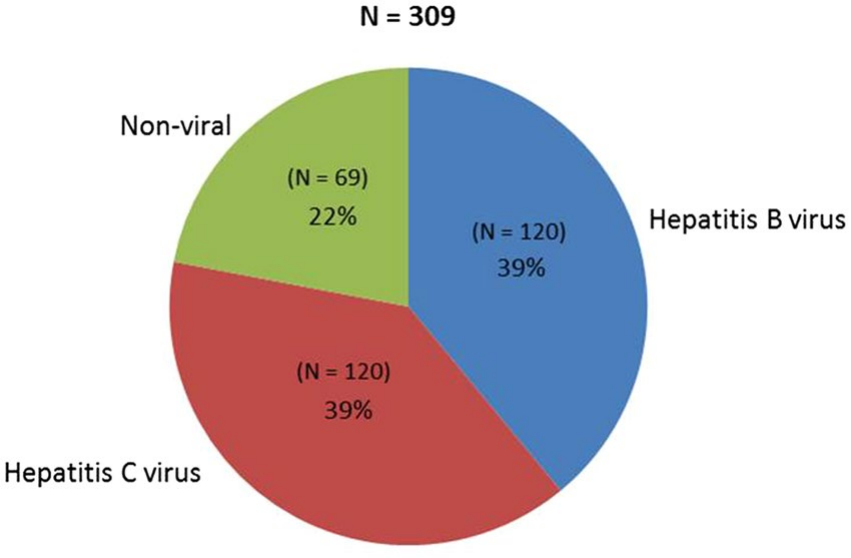
Liver disease etiology for patients without hepatocellular carcinoma.

**Figure 2:**
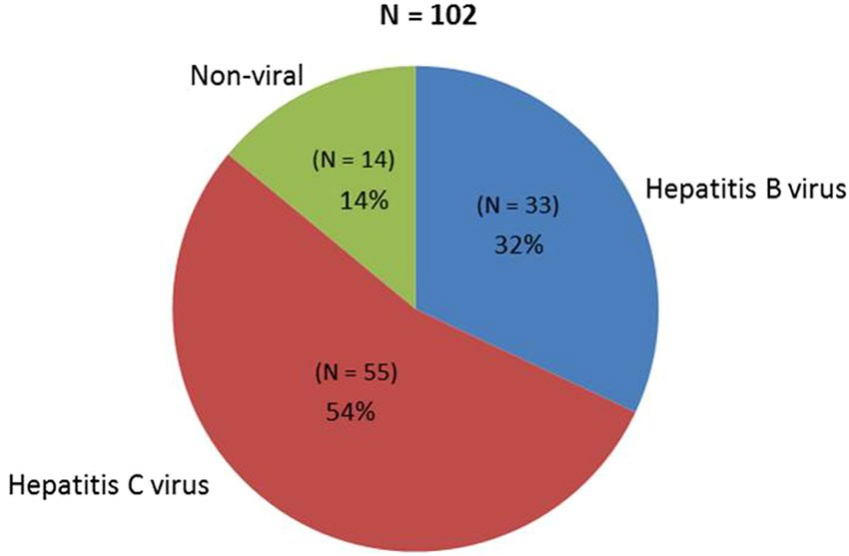
Liver disease etiology for patients with hepatocellular carcinoma.

Of the 411 patients in our study, 102 had HCC and 309 did not. Among the 309 non-HCC patients, 120 had chronic hepatitis B, 120 had chronic hepatitis C and 69 had non-viral liver disease (Fig. 1). Among the 102 HCC patients, most had chronic hepatitis C (55 patients), followed by chronic hepatitis B (33 patients) and non-viral liver disease (14 patients) (Fig. 2).

Most of the non-HCC patients with a viral etiology were male (61%). Among the chronic hepatitis B non-HCC patients (“HBV non-HCC”), most were Asian (89%), while the chronic hepatitis C non-HCC (“HCV non-HCC”) patients were mostly Caucasian (59%). Compared with HBV non-HCC patients, HCV non-HCC patients were more likely to be younger, have a history of alcohol and tobacco use, cirrhosis, decompensation symptoms, Child’s class A and B, and prior interferon therapy (Table 1). None of the chronic hepatitis C patients received direct-acting antiviral therapy.

**Table 1:** Characteristics of patients without hepatocellular carcinoma (HCC).

Characteristic	Chronic Hepatitis B without HCC N = 120	Chronic Hepatitis C without HCC N = 120	*P* value
**Gender and Age**	N = 120	N = 120	
Male	76 (63.3%)	70 (58.3%)	0.43
Age	47.3 ± 12.6	53.6 ± 10.2	<0.0001
**Ethnicity**	N = 120	N = 120	
Asian	107 (89.2%)	17 (14.2%)	<0.0001
Caucasian	10 (8.3%)	71 (59.2%)	<0.0001
Hispanic	0 (0%)	24 (20.0%)	<0.0001
African American	0 (0%)	3 (2.5%)	0.08
Other	3 (2.5%)	5 (4.2%)	0.47
**Habits**	N = 117	N = 98–104	
History of Alcohol Use	5 (4.3%)	44 (44.9%)	<0.0001
History of Tobacco Use	29 (24.8%)	73 (70.2%)	<0.0001
**Cirrhosis Status**	N = 120	N = 120	
Baseline Cirrhosis	29 (24.2%)	102 (85.0%)	<0.0001
**Hepatic Decompensation**	N = 120	N = 120	
Any Decompensation	17 (14.2%)	88 (73.3%)	<0.0001
Ascites	12 (10.0%)	63 (52.5%)	<0.0001
Hepatic Encephalopathy	5 (4.2%)	62 (51.7%)	<0.0001
Variceal Bleeding	5 (4.2%)	15 (12.5%)	0.02
**Classification and Scoring**	N = 14–15	N = 69–71	
Average MELD	9.8 ± 2.8	12.2 ± 5.1	0.08
Child’s Class A	12 (85.7%)	31 (44.9%)	0.01
Child’s Class B	1 (7.1%)	28 (40.6%)	0.02
Child’s Class C	1 (7.1%)	10 (14.5%)	0.46
**Prior Treatment**	N = 120	N = 117	
Any Prior Interferon Therapy	4 (3.3%)	48 (41.0%)	<0.0001
Any Prior Nucleos(t)ide Therapy	69 (57.5%)	44 (35.9%)	0.001

Comparison of characteristics of patients without HCC with chronic hepatitis B vs. chronic hepatitis C.

Among HCC patients, chronic hepatitis C patients were more likely to be non-Asian, have hepatic encephalopathy, Child’s class A and B, and prior interferon therapy. There was no difference in age, alcohol or tobacco use, tumor characteristics, MELD scores, or prior HCC treatment (Table 2). A total of 22 patients received chemotherapy as treatment for their HCC, and none of the patients received immune-based therapies, such as check-point inhibitors.

**Table 2:** Characteristics of patients with hepatocellular carcinoma (HCC).

Characteristic	Chronic Hepatitis B with HCC N = 33	Chronic Hepatitis C with HCC N = 55	*P* value
**Gender and Age**	N = 33	N = 55	
Male	28 (84.9%)	45 (81.8%)	0.71
Age	59.1 ± 11.2	63.1 ± 9.9	0.09
**Ethnicity**	N = 33	N = 55	
Asian	30 (90.9%)	29 (52.7%)	<0.0001
Caucasian	3 (9.1%)	16 (29.1%)	0.03
Hispanic	0 (0%)	8 (14.6%)	0.02
African American	0 (0%)	1 (1.8%)	0.44
Other	0 (0%)	1 (1.8%)	0.44
**Habits**	N = 27–29	N = 44–48	
History of Alcohol Use	9 (33.3%)	16 (36.4%)	0.80
History of Tobacco Use	16 (55.2%)	30 (62.5%)	0.53
**Cirrhosis Status**	N = 33	N = 55	
Baseline Cirrhosis	29 (87.9%)	51 (92.7%)	0.44
**Hepatic Decompensation**	N = 33	N = 55	
Any Decompensation	28 (84.9%)	45 (81.8%)	0.71
Ascites	24 (72.7%)	36 (65.5%)	0.48
Hepatic Encephalopathy	4 (12.1%)	20 (36.4%)	0.01
Variceal Bleeding	1 (3.0%)	5 (9.1%)	0.28
**Classification and Scoring**	N = 18–19	N = 39–41	
Average MELD	9.5 ± 3.3	10.1 ± 3.5	0.57
Child’s Class A	16 (88.9%)	25 (64.1%)	0.05
Child’s Class B	1 (5.6%)	13 (33.3%)	0.02
Child’s Class C	1 (5.6%)	1 (2.6%)	0.57
**Tumor Characteristics**	N = 22–33	N = 38–49	
Unifocal Tumor	16 (72.7%)	25 (65.8%)	0.58
Tumor Size Average	5.5 ± 4.0	4.9 ± 3.8	0.56
Vascular Invasion	3 (9.1%)	4 (8.2%)	0.88
Within Milan Criteria	10 (43.5%)	21 (55.3%)	0.37
**Prior Treatment**	N = 33	N = 55	
Any Prior Interferon Therapy	1 (3.0%)	14 (25.5%)	0.01
Any Prior Nucleos(t)ide Therapy	20 (60.6%)	11 (20.0%)	<0.0001
Prior HCC Treatment	14 (42.4%)	16 (29.1%)	0.20

Comparison of characteristics of HCC patients with chronic hepatitis B vs. chronic hepatitis C.

### Serum Cytokine Profile Comparison among non-HCC Patients

Serum cytokine profiles of patients who did not have HCC (“non-HCC”) were compared based on etiology and cirrhosis status.

**Figure 3:**
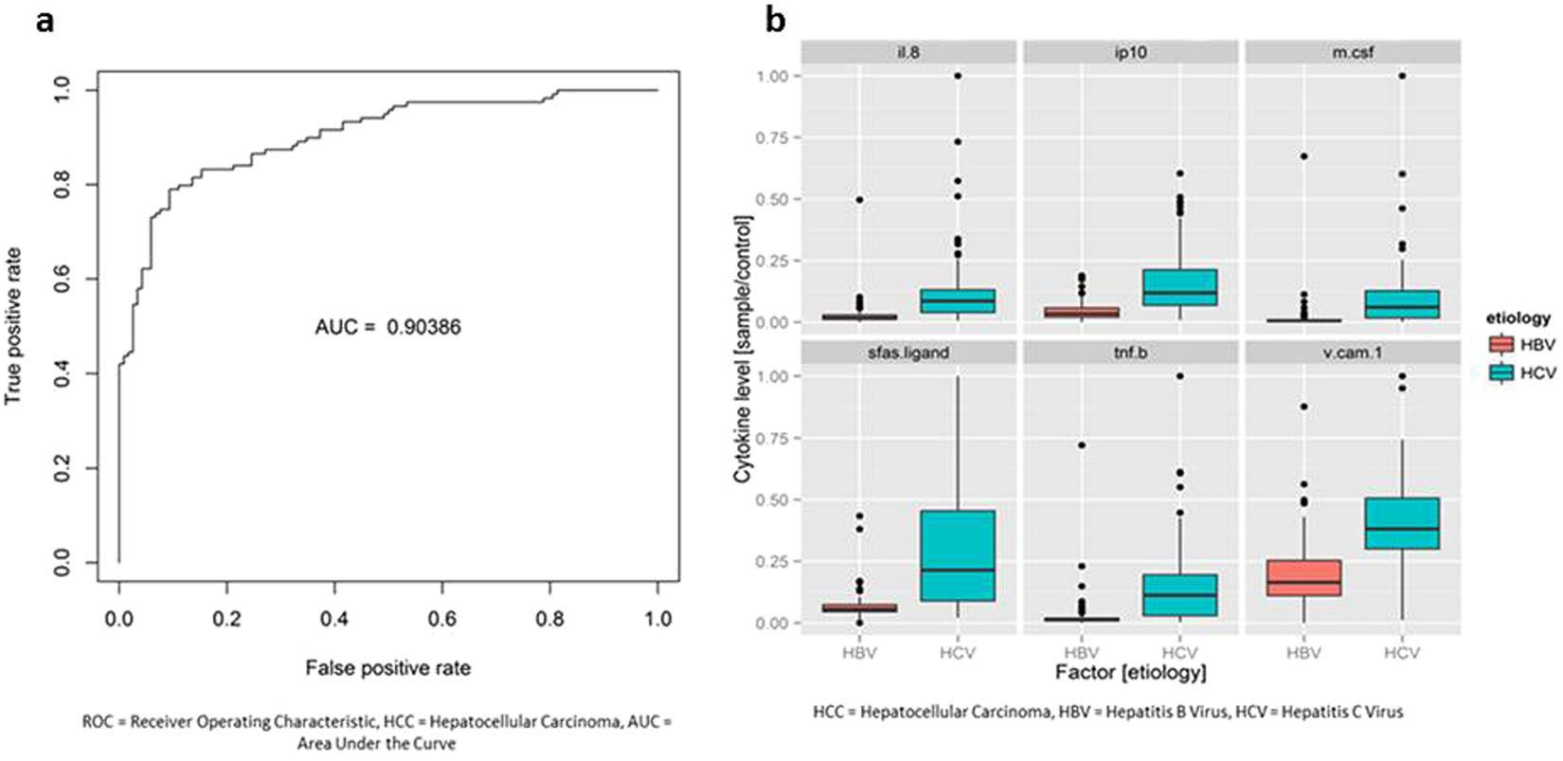
Serum cytokine and chemokine profile comparison of patients without hepatocellular carcinoma (non-HCC) with chronic hepatitis B vs. non-HCC patients with chronic hepatitis C. (**a**) Receiver operating characteristic (ROC) plot with area under the curve (AUC). (**b**) Levels of top predictive cytokines.

**Figure 4:**
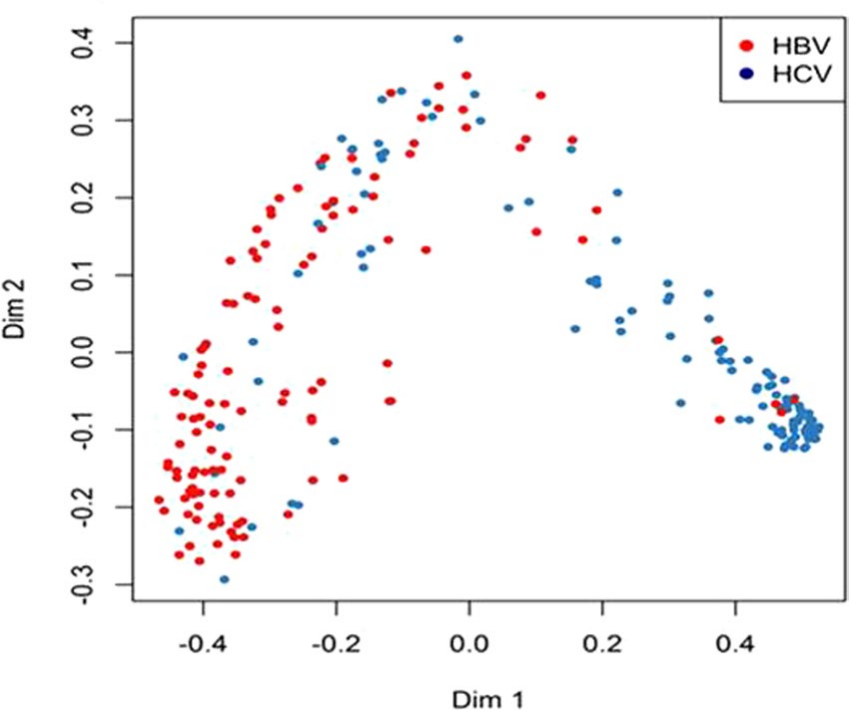
Multidimensional scaling plot comparing serum cytokine profiles of non-hepatocellular carcinoma (HCC) patients with chronic hepatitis B (red dots) vs. non-HCC patients with chronic hepatitis C (blue dots).

Serum cytokine profiles of non-HCC patients with chronic hepatitis B (n = 120) vs. chronic hepatitis C (n = 120) differed substantially with respect to the area under the curve of the receiver operating characteristic plot (AUROC = 0.904) (Fig. 3a). Top predictive cytokines were sFas ligand, M-CSF, TNF-β, IP-10, IL-8, and VCAM-1 (Fig. 3b, see Supplementary Fig. S1). Additional comparison using multidimensional scaling plots also showed a clear distinction between patients with chronic hepatitis B vs. C (Fig. 4). In addition, serum cytokine profiles were different between patients with chronic hepatitis B and chronic hepatitis C both in those with (AUROC = 0.820) and without cirrhosis (AUROC = 0.803). Among Asians without HCC, there was a significant difference between those with chronic hepatitis B and C (AUROC = 0.859, see Supplementary Fig. S2), but there was no difference between Asians and non-Asians among patients with chronic hepatitis B (AUROC = 0.512) or chronic hepatitis C (AUROC = 0.548).

Furthermore, cytokine profiles in chronic hepatitis B non-HCC patients differed from those of patients with non-viral disease with AUROC = 0.840 (see Supplementary Fig. S3) with leptin, resistin, IL-8, M-CSF, TNF-β, and sFas ligand as the top 6 cytokines with distinguishing levels. Comparison between non-HCC HCV (n = 120) and non-HCC non-viral (n = 69) also showed a differentiating pattern though not as pronounced (AUROC = 0.720) (see Supplementary Fig. S4).

### Serum Cytokine Profile Comparison among HCC Patients

**Figure 5:**
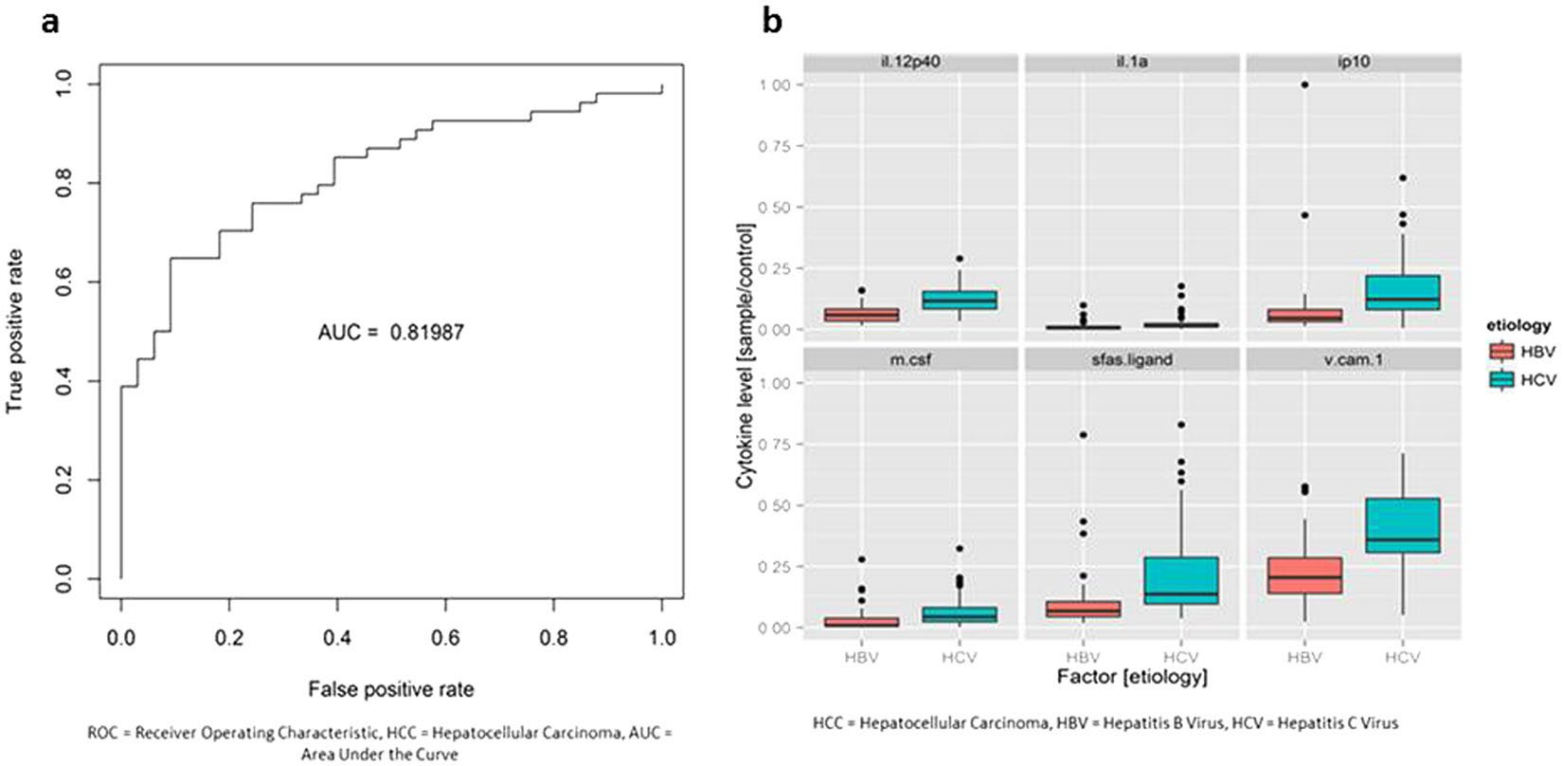
Serum cytokine and chemokine profile comparison of patients with chronic hepatitis B with hepatocellular carcinoma (HCC) vs. chronic hepatitis C with HCC. (**a**) Receiver operating characteristic (ROC) plot with area under the curve (AUC). (**b**) Levels of top predictive cytokines.

**Figure 6:**
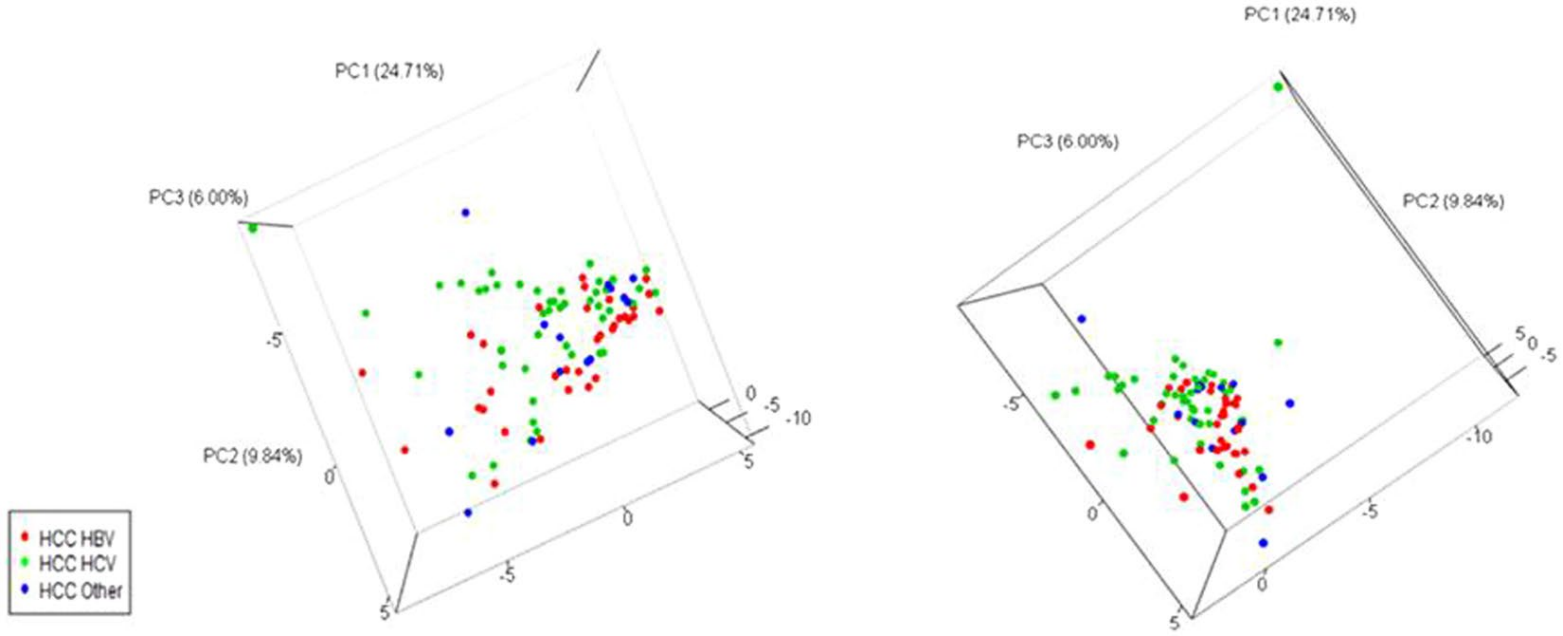
Serum cytokine levels of hepatocellular carcinoma (HCC) patients with chronic hepatitis B, C, or non-viral disease. Three-dimensional principal component analysis (PCA) plot comparing serum cytokine profiles of HCC patients with chronic hepatitis B (red dots) vs. C (green dots) vs. non-viral (blue dots).

Among the patients with HCC, we compared those with chronic hepatitis B (n = 33) vs. those with chronic hepatitis C (n = 55) and found significant differences in their cytokine profiles (AUROC = 0.820) (Fig. 5a). The top predictive cytokines were IP-10, IL-12 p40, VCAM-1, IL-1α, sFas ligand, and M-CSF (Fig. 5b, see Supplementary Fig. S5). However, unlike patients without HCC, there was no appreciable difference in serum cytokine profiles of HCC patients with chronic hepatitis B or C compared to non-viral HCC patients. Specifically, analysis of HCC patients with chronic hepatitis B (n = 33) vs. non-viral etiologies (n = 14) showed an AUROC of only 0.526, and analysis of HCC patients with chronic hepatitis C (n = 55) vs. non-viral etiologies (n = 14) showed a similar AUROC of 0.509. PCA 3-dimensional plots comparing serum cytokine levels in HCC patients based on etiology shows a visible distinction between HBV-related HCC and HCV-related HCC, but non-viral HCC could not be distinguished from HBV-HCC or HCV-HCC (Fig. 6).

### Serum Cytokine Profile Comparison between HCC and non-HCC Patients

**Figure 7:**
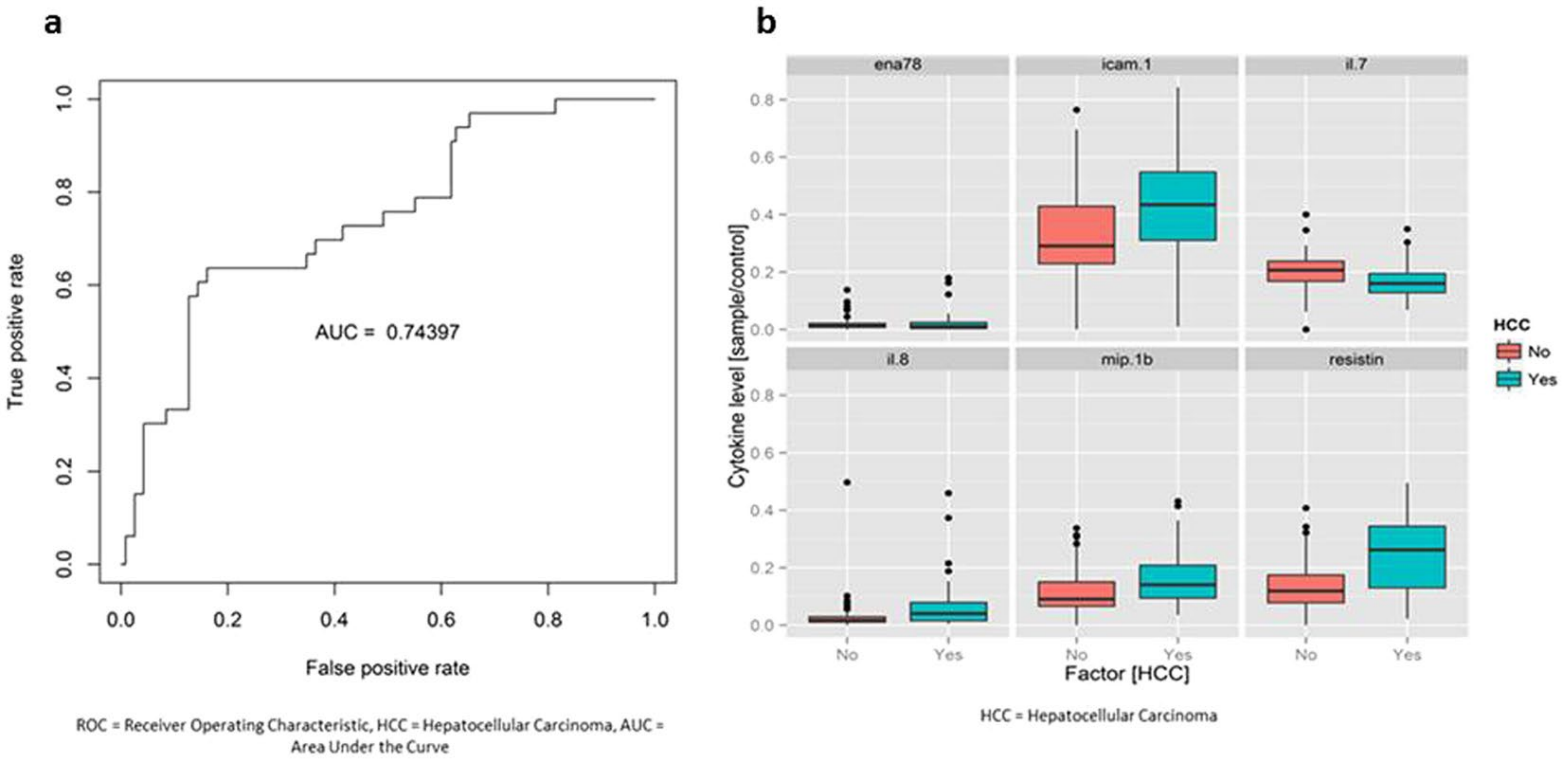
Serum cytokine and chemokine profile comparison of chronic hepatitis B patients with vs. without hepatocellular carcinoma (HCC). (**a**) Receiver operating characteristic (ROC) plot with area under the curve (AUC). (**b**) Levels of top predictive cytokines.

In patients with chronic hepatitis B, analysis of cytokine profiles of HCC patients (n = 33) vs. non-HCC patients (n = 120) revealed an AUROC of 0.744 (Fig. 7a). The top six biomarkers that contributed to this differentiation were resistin, IL-7, IL-8, ENA-78, ICAM-1, and MIP-1b (Fig. 7b, see Supplementary Fig. S6). The HCC patients were more likely to be in the reactivation phase compared to the non-HCC patients, but there was no difference in the other phases of HBV infection (Table 3). Analysis comparing cirrhotic HBV patients with HCC (n = 29) vs cirrhotic HBV patients without HCC (n = 29) had an AUROC of only 0.503 (Supplementary Fig. S7).

**Table 3:** Phases of hepatitis B virus infection.

Phases of Hepatitis B Virus Infection	Chronic Hepatitis B without HCC N = 120	Chronic Hepatitis B with HCC N = 33
HBeAg-positive Immune Tolerant	8 (7.0%)	0 (0%)
HBeAg-positive Immune Active	9 (7.9%)	2 (8.0%)
HBeAg-negative Inactive Carrier	53 (46.5%)	9 (36.0%)
HBeAg-negative Immune Active	13 (11.4%)	19 (76.0%)

Comparison of the four hepatitis B virus infection phases between chronic hepatitis B patients with hepatocellular carcinoma and without hepatocellular carcinoma.

**Figure 8:**
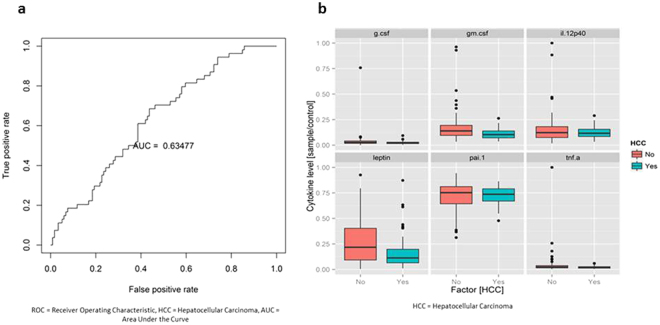
Serum cytokine and chemokine profile comparison of patients with chronic hepatitis C with hepatocellular carcinoma (HCC) vs. without HCC. (**a**) Receiver operating characteristic (ROC) plot with area under the curve (AUC). (**b**) Levels of top predictive cytokines.

Among chronic hepatitis C patients, analysis of HCC (n = 55) and non-HCC patients (n = 120) yielded an AUROC of 0.634 (Fig. 8a). Top six predictive cytokines were G-CSF, GM-CSF, IL12p40, leptin, PAI-1, TNF-α (Fig. 8b, see Supplementary Fig. S8). There were 69 chronic hepatitis C patients with genotype 1, 13 with genotype 2, 15 with genotype 3, 1 with genotype 4, and 4 with genotype 6. Between non-HCC and HCC cases, there was no significant difference in HCV RNA levels or HCV genotypes, except 1% of non-HCC cases and 12% of HCC cases had genotype 6 (p = 0.02).

## Discussion

The results of this study demonstrated a clear difference in serum cytokine profiles between patients with chronic hepatitis B vs. C, chronic hepatitis B vs. non-viral liver disease, and chronic hepatitis C vs. non-viral liver disease. Similarly, there was a significant difference among HCC cases based on viral etiology, but there was no difference between viral and non-viral etiology. Comparison of cytokine profiles from chronic hepatitis B patients with vs. without HCC were significantly different. However, comparison of cirrhotic HBV patients with vs. without HCC did not show a significant difference. Thus, the observed cytokine profile differences between the overall HBV HCC and non-HCC cohorts may have been due to inflammation and cirrhosis and not due to the presence of HCC. Similarly, the distinction was not clear for chronic hepatitis C patients with vs. without HCC.

Among patients who did not have HCC, cytokine profiling accurately differentiated patients with chronic hepatitis B from those with chronic hepatitis C (AUROC = 0.904) and non-viral etiologies (AUROC = 0.840). Cytokine profiling also showed different patterns between patients with chronic hepatitis C and non-viral disease (AUC = 0.720). The difference between chronic hepatitis B and C patients persisted in separate sub-analyses of patients with cirrhosis (AUROC = 0.820) and those without cirrhosis (AUROC = 0.803). Previous studies have also noted a difference in cytokine levels between HBV and HCV infections^14,15^; however, there is no consensus on which cytokines are the most important for disease progression and HCC carcinogenesis. Some studies comparing patients with hepatitis B and C showed increased levels of plasma IFN-γ, TNF-α, and IL-2 in patients with hepatitis B^14^. Our results showed sFas-ligand, M-CSF, TNF-β, IP-10, and IL-8 as the top 5 cytokines with highest predictive power when comparing HBV vs. HCV etiologies, a different set of cytokines. Additionally, sFas-ligand, TNF-β, and IL-8 were not identified in another study^24^, possibly because they included only acute hepatitis patients, while we studied chronic hepatitis patients.

In regards to differences seen in comparison of HCC cases based on viral etiology (HBV-HCC vs. HCV-HCC, AUC = 0.820), the findings are likely due to HBV’s direct and HCV’s indirect pathways for HCC development. There was no difference in prior HCC treatment between patients with chronic hepatitis B and C, and only a small number of patients (n = 22) received chemotherapy for their HCC. Thus, prior HCC treatment would not have had a significant impact on cytokine levels, and the sample size was too small to perform a subanalysis. Comparison of cytokine profiles of HBV-HCC and HCV-HCC patients in our study yielded IP-10 and IL-12p40 as the top predictive cytokines with higher levels of these biomarkers in HCV-HCC cases. In addition, several studies have found increased levels of IP-10 in HCC patients^23^ and have compared levels of IP-10 between HBV non-HCC and HCV non-HCC patients^25^, but few have studied IP-10 levels based on etiology in HCC patients. We also found a higher concentration of IL-12p40 in HCV-HCC cases. IL-12p40 is one of the subunits of IL-12 and IL-23^26,27^. There is a paucity of data on IL-12p40’s mechanistic role in HCC development; however, prior studies have shown that IL-12 and IL-23 are important in HCC pathogenesis. IL-12 can inhibit the growth of HCC^28^, while IL-23 helps promote tumor growth in mice^29^. Another study done on human HCC samples also found that IL-23 promotes metastasis via the NF-kB/p65 signaling pathway^30^. While several of these studies provide a mechanistic role for these cytokines, they did not make a distinction between etiologies of HCC. Our results suggest that IP-10 and IL-12p40 may be more important in progression to HCV-related HCC compared to HBV-related HCC.

Although we found cytokine profile differences between the viral etiologies among HCC patients, there was no difference between the viral and non-viral etiologies (HBV-HCC vs. non-viral HCC AUC = 0.526; HCV-HCC vs. non-viral HCC AUC = 0.509). The non-viral HCC cohort were a heterogeneous group of cases with a variety of non-viral etiologies with varying cytokine profiles, some of which could be similar to HBV profiles and others similar to HCV profiles, thereby hindering detection of a clear overall difference. Therefore, cytokine profiles of non-viral HCC should be further subdivided into the various specific non-viral liver diseases and individually studied to see if there is a difference in their cytokine profiles compared to profiles of viral etiology cases.

When comparing HCC and non-HCC cases, profiles of HCC and non-HCC chronic hepatitis B patients were rather different with resistin being the most predictive cytokine followed by IL-7 and IL-8 (AUC = 0.744) and less so for chronic hepatitis C patients (AUC = 0.635). Compared to non-HCC patients, our patients with HBV-HCC had higher levels of resistin and IL-8 and lower levels of IL-7. The higher level of resistin in HCC cases may be consistent with prior studies reporting the role of resistin in promoting cellular metastasis by inducing ICAM-1 and VCAM-1, which can hypothetically allow the HCC cells to adhere to vascular endothelium^31^. There is also evidence that IL-8 can promote angiogenesis^32^ and metastasis^33^ *in vitro*. On the other hand, there is limited data on IL-7’s role in pathogenesis of HBV-related HCC. Because our study showed lower levels of IL-7 in patients with HBV-HCC, loss of IL-7 activity may be a component of HCC development. Although the above mentioned studies reported mechanistic roles for resistin and IL-8 in HCC progression, these experiments were not performed on HCC specifically due to hepatitis B. Additionally, more HCC patients were in the reactivation phase compared to non-HCC patients, which could have affected cytokine levels. Cirrhosis could also have affected cytokine levels given that analysis comparing cirrhotic HBV patients with HCC vs. without HCC was not significant, though sample sizes are small for both groups. Future studies with larger sample sizes comparing cirrhotic HBV with and without HCC (we only had 29 patients in each group) as well as non-cirrhotic HBV patients with vs. without HCC (we only had 4 non-cirrhotic HBV-related HCC cases) are needed to clarify these results. Additionally, comparison groups should also be stratified by HBeAg and hepatitis activity status. Our results can guide future research on HCC pathogenesis and metastasis in patients with HBV-related HCC.

One of the limitations of this study is the distribution of race among the etiologies. Most of our HBV patients were Asian, whereas most of our HCV patients were non-Asian. Knowledge of liver disease progression among different ethnic groups is still poorly understood. Moreover, very little is known about cytokine differences based on ethnicity, and no study to date has examined this in regards to potential ethnic differences in patients with viral hepatitis or HCC. Therefore, it would be difficult to generalize our findings to all ethnicities. However, we still observed a significant difference in cytokine profiles among non-HCC Asian patients with chronic hepatitis B vs. C. In addition, Asian ethnicity did not seem to affect cytokine differences since there was no difference between Asians and non-Asians among chronic hepatitis B or C cases. Another limitation was the limited analysis for the non-viral cohort and for the cohort of HBV patients without cirrhosis given their smaller sample size. Furthermore, differences in prior hepatitis treatment with interferon or nucleos(t)ide analogs may have had an effect on cytokine levels of non-HCC cases, and previous studies have seen some cytokine levels change after hepatitis therapy (i.e. TNF-α, IL-8, sFas ligand, beta 2-microglobulin, neopterin, and soluble IL-2 receptor)^34–38^. Finally, the cross-sectional nature of this study raises the possibility that the presence of liver disease led to changes in cytokine levels, and that observed serum cytokine profiles are not representative of pre-disease, risk-related patterns.

## Conclusion

In conclusion, HBV-related HCC and HCV-related HCC are two different diseases, not just based on their clinical presentation, but on their serum cytokine profiles, as well. Our study also showed that, despite both being viral diseases and both associated with chronic inflammation, HBV and HCV have highly distinct serum cytokine patterns. Cytokine profiles did not distinguish cirrhotic HBV patients with and without HCC (AUC 0.503) or HCV patients with and without HCC (AUC 0.63).

## Electronic supplementary material

Supplementary Information
