# Supplementary material for: Differential Serum Cytokine Profiles in Patients with Chronic Hepatitis B, C, and Hepatocellular Carcinoma
Source: Sci Rep. 2017 Sep 19;7:11867. doi: 10.1038/s41598-017-11975-7 (PMC5605527; doi:10.1038/s41598-017-11975-7)
Supplement: Supplementary file 1 — Supplementary Information [file 41598_2017_11975_MOESM1_ESM.pdf]

Supplementary Information

Differential Serum Cytokine Profiles in Patients with Chronic Hepatitis B, C, and Hepatocellular Carcinoma

Jacqueline Estevez[1,2], Vincent L. Chen[3,4], Ondrej Podlaha[5], Biao Li[5], An Le[1], Philip Vutien[1,6], Ellen T. Chang[7], Yael Rosenberg-Hasson[8], Zhaoshi Jiang[5], Stefan Pflanz[5], Dongliang Ge[5], Anuj Gaggar[5], and Mindie H. Nguyen[1]

[1]Stanford University Medical Center, Division of Gastroenterology and Hepatology, Stanford, CA 94305, USA

[2]Boston University School of Medicine, Boston, MA 02118, USA

[3]Stanford University Medical Center, Department of Medicine, Stanford, CA 94305, USA

[4]University of Michigan, Division of Gastroenterology and Hepatology, Ann Arbor, MI, USA

[5]Gilead Sciences, Foster City, CA 94404, USA

[6]Rush University Medical Center, Chicago, IL 60612, USA

[7]Stanford University School of Medicine, Department of Health Research and Policy (Epidemiology), Stanford, CA 94305, USA

[8]Stanford University Medical Center, The Human Immune Monitoring Center, Stanford, CA 94305, USA

Top predictive biomarkers for serum cytokine profiles of non-hepatocellular carcinoma patients with chronic hepatitis B vs. chronic hepatitis C

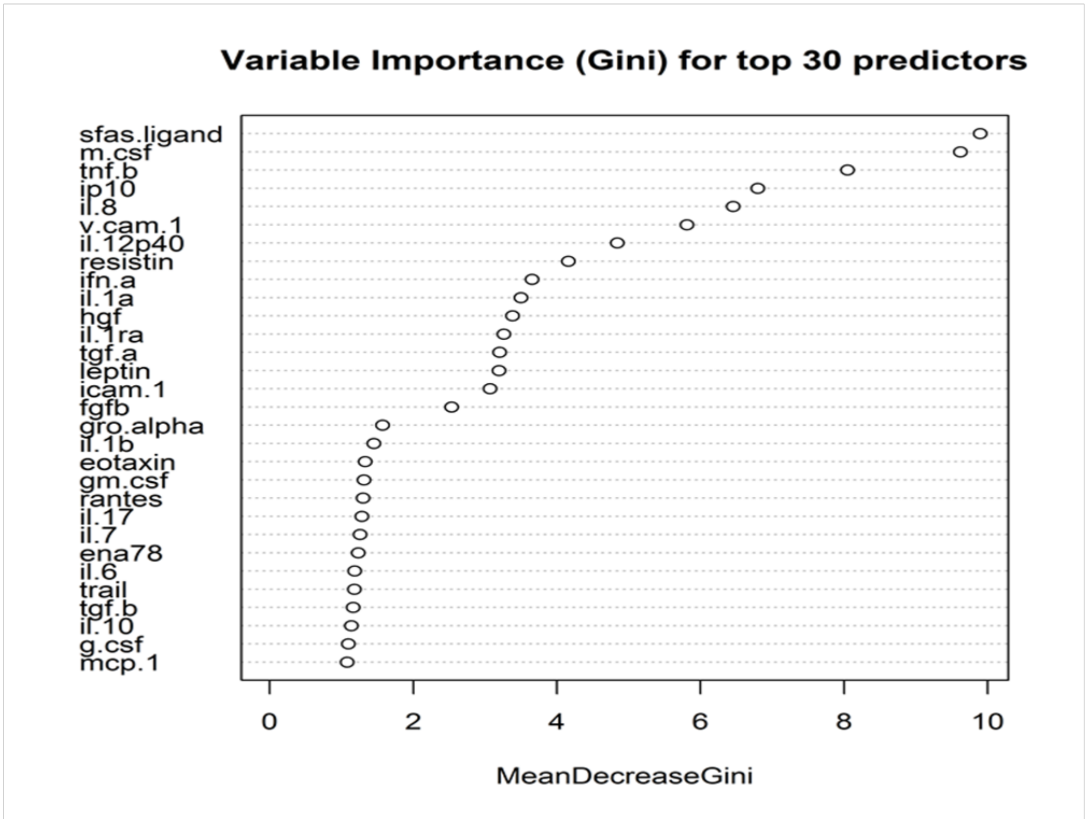

Supplemental Figure S1. Gini graph of top predictive cytokines and chemokines of non-hepatocellular carcinoma patients with chronic hepatitis B vs. chronic hepatitis C.

Serum cytokine profiles of non-hepatocellular carcinoma Asian patients with chronic hepatitis B vs. chronic hepatitis C

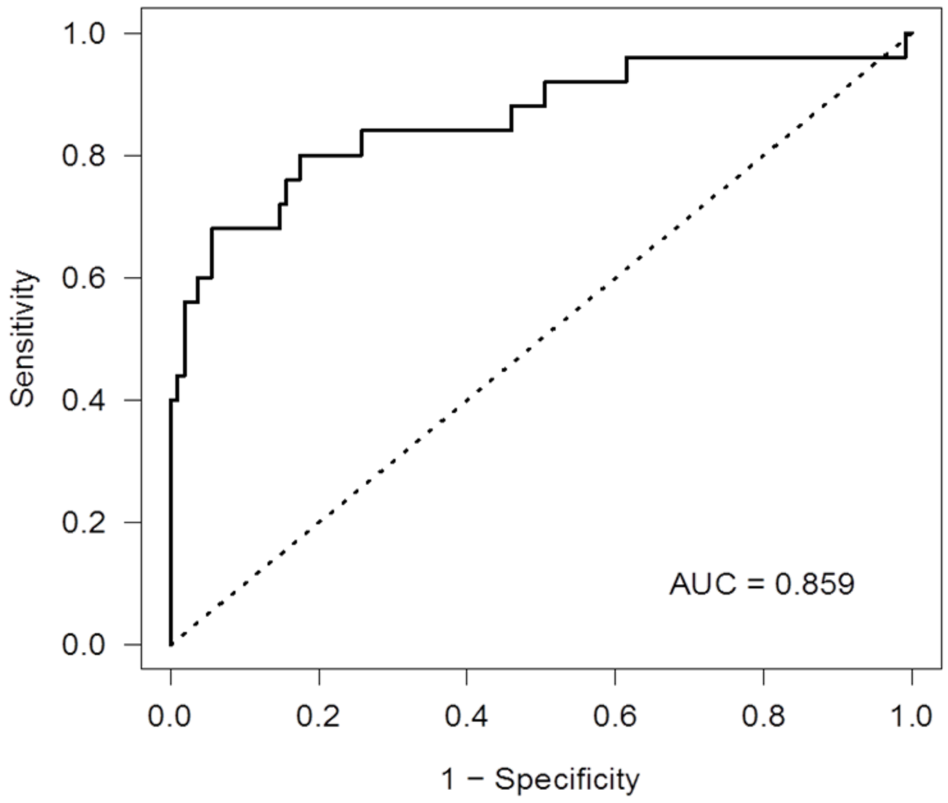

Supplemental Figure S2. Receiver operating characteristic plot with area under the curve (AUC) comparing serum cytokine and chemokine profiles of Asian patients without hepatocellular carcinoma with chronic hepatitis B vs. chronic hepatitis C.

Serum cytokine profiles of non-hepatocellular carcinoma patients with chronic hepatitis B vs. non-viral hepatitis

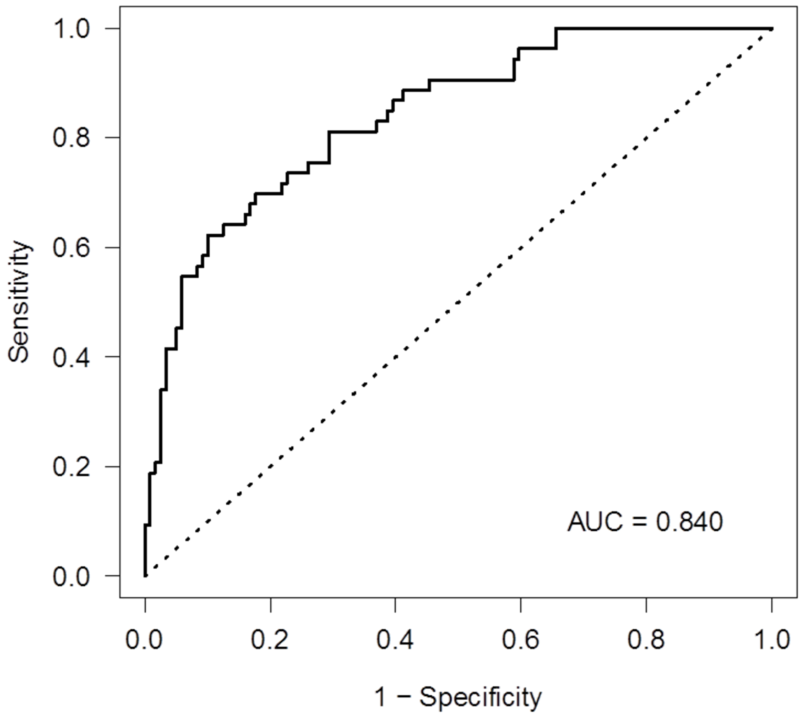

Supplemental Figure S3. Receiver operating characteristic plot with area under the curve (AUC) comparing serum cytokine and chemokine profiles of patients without hepatocellular carcinoma with chronic hepatitis B vs. non-viral hepatitis.

Serum cytokine profiles of non-hepatocellular carcinoma patients with chronic hepatitis C vs. non-viral hepatitis

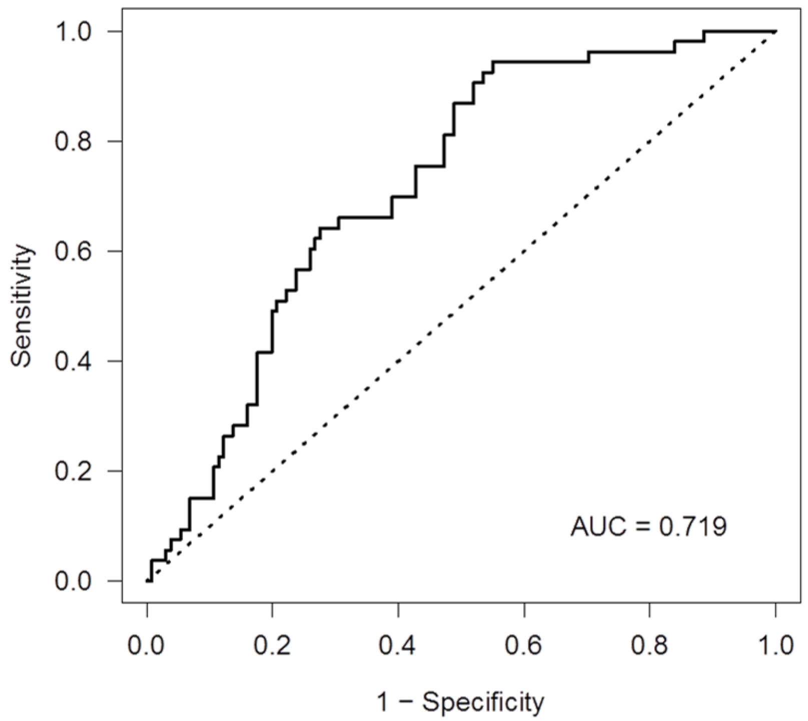

Supplemental Figure S4. Receiver operating characteristic plot with area under the curve (AUC) comparing serum cytokine and chemokine profiles of patients without hepatocellular carcinoma with chronic hepatitis C vs. non-viral hepatitis.

Top predictive biomarkers for serum cytokine profiles of hepatocellular carcinoma patients with chronic hepatitis B vs. chronic hepatitis C

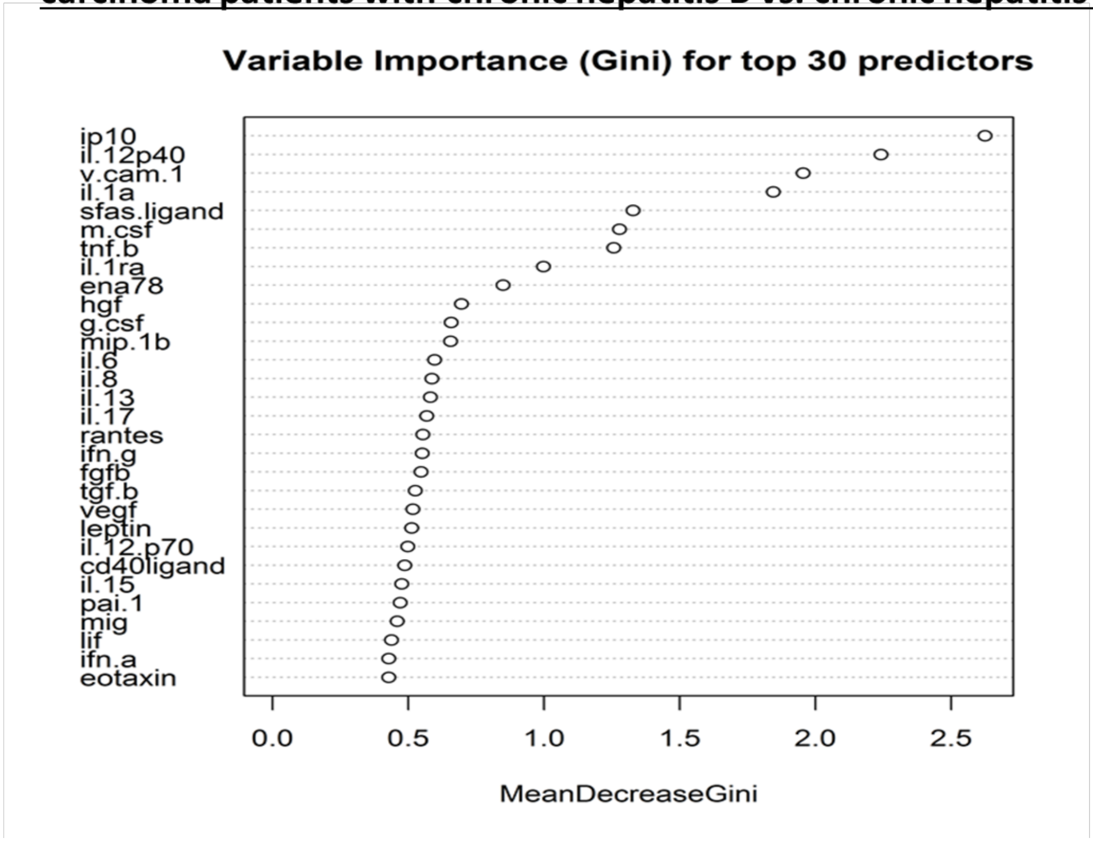

Supplemental Figure S5. Gini graph for top predictive cytokines and chemokines of hepatocellular carcinoma patients with chronic hepatitis B vs. chronic hepatitis C.

Serum cytokine profiles of chronic hepatitis B patients with vs. without hepatocellular carcinoma

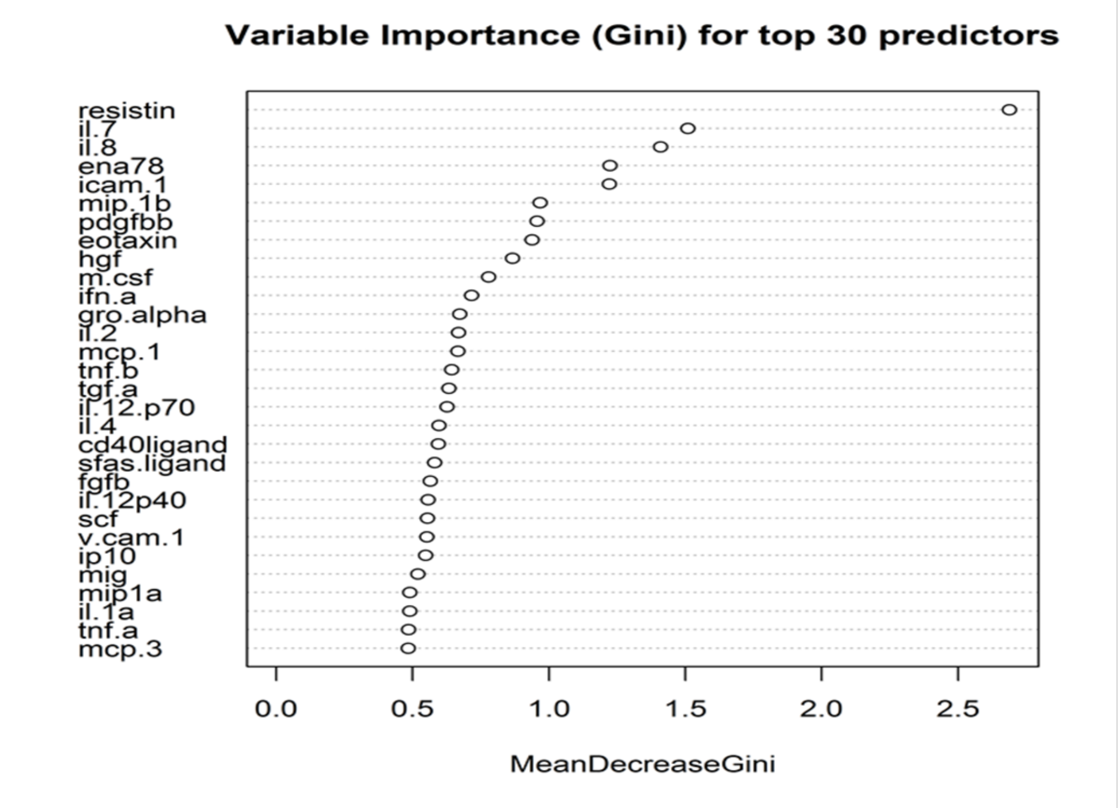

Supplemental Figure S6. Gini graph for top predictive cytokines and chemokines of chronic hepatitis B patients with vs. without hepatocellular carcinoma.

Serum cytokine profiles of cirrhotic chronic hepatitis B patients with hepatocellular carcinoma vs. no hepatocellular carcinoma

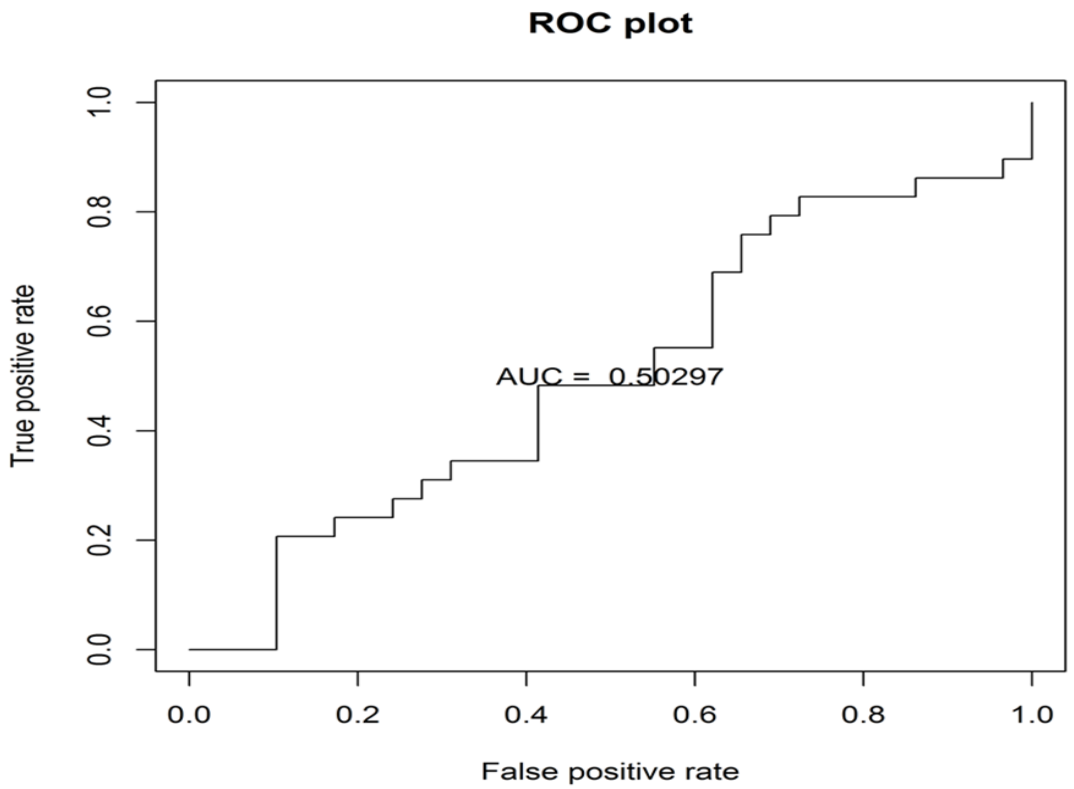

Supplemental Figure S7. Receiver operating characteristic plot with area under the curve (AUC) comparing serum cytokine and chemokine profiles of cirrhotic chronic hepatitis B patients with hepatocellular carcinoma vs. without hepatocellular carcinoma.

Serum cytokine profiles of chronic hepatitis C patients with vs. without hepatocellular carcinoma

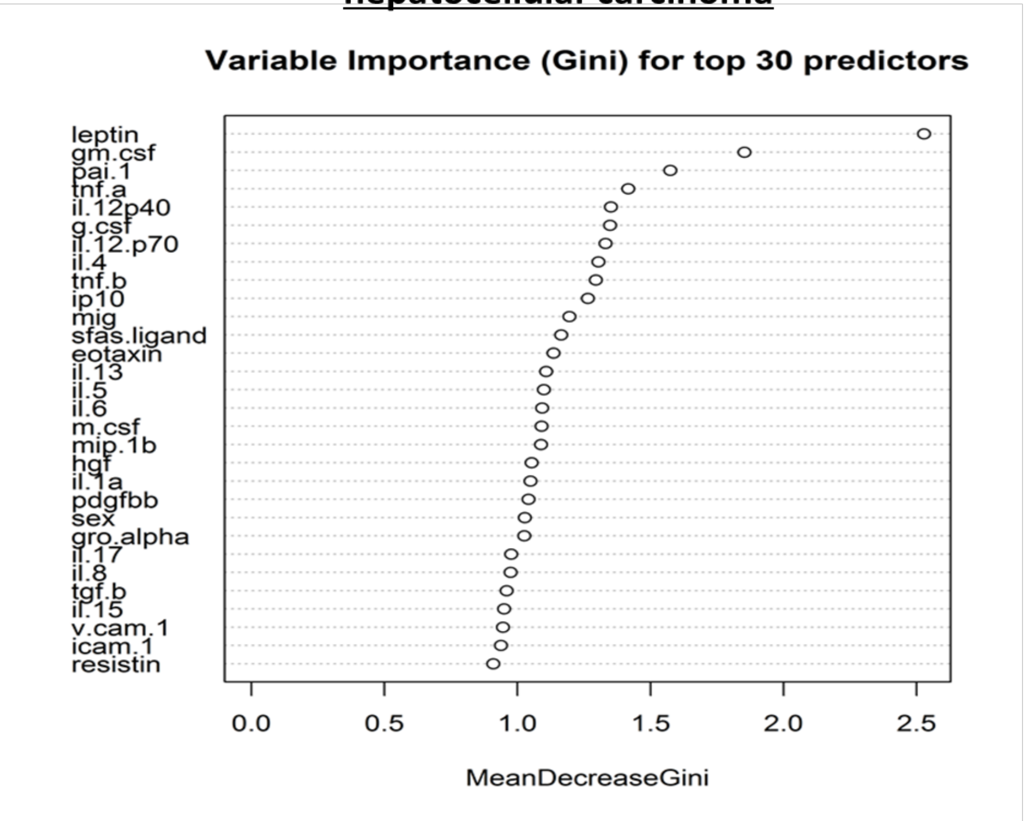

Supplemental Figure S8. Gini graph for top predictive cytokines and chemokines of chronic hepatitis C patients with vs. without hepatocellular carcinoma.
